# Performance of Mapping Approaches for Whole-Genome Bisulfite Sequencing Data in Crop Plants

**DOI:** 10.3389/fpls.2020.00176

**Published:** 2020-02-28

**Authors:** Claudius Grehl, Marc Wagner, Ioana Lemnian, Bruno Glaser, Ivo Grosse

**Affiliations:** ^1^ Institute of Computer Science, Bioinformatics, Martin Luther University Halle–Wittenberg, Von Seckendorff-Platz 1, Halle (Saale), Germany; ^2^ Institute of Agronomy and Nutritional Sciences, Soil Biogeochemistry, Martin Luther University Halle–Wittenberg, Von Seckendorff-Platz 3, Halle (Saale), Germany; ^3^ Institute of Mathematics and Informatics, Freie Universität Berlin, Berlin, Germany; ^4^ Institute of Human Genetics, Martin Luther University Halle-Wittenberg, Halle (Saale), Germany; ^5^ Bioinformatics Unit, German Centre for Integrative Biodiversity Research (iDiv) Halle-Jena-Leipzig, Leipzig, Germany

**Keywords:** epigenetics, DNA methylation patterns, read mapping, benchmarking, WGBS

## Abstract

DNA methylation is involved in many different biological processes in the development and well-being of crop plants such as transposon activation, heterosis, environment-dependent transcriptome plasticity, aging, and many diseases. Whole-genome bisulfite sequencing is an excellent technology for detecting and quantifying DNA methylation patterns in a wide variety of species, but optimized data analysis pipelines exist only for a small number of species and are missing for many important crop plants. This is especially important as most existing benchmark studies have been performed on mammals with hardly any repetitive elements and without CHG and CHH methylation. Pipelines for the analysis of whole-genome bisulfite sequencing data usually consists of four steps: read trimming, read mapping, quantification of methylation levels, and prediction of differentially methylated regions (DMRs). Here we focus on read mapping, which is challenging because un-methylated cytosines are transformed to uracil during bisulfite treatment and to thymine during the subsequent polymerase chain reaction, and read mappers must be capable of dealing with this cytosine/thymine polymorphism. Several read mappers have been developed over the last years, with different strengths and weaknesses, but their performances have not been critically evaluated. Here, we compare eight read mappers: Bismark, BismarkBwt2, BSMAP, BS-Seeker2, Bwameth, GEM3, Segemehl, and GSNAP to assess the impact of the read-mapping results on the prediction of DMRs. We used simulated data generated from the genomes of *Arabidopsis thaliana*, *Brassica napus*, *Glycine max*, *Solanum tuberosum*, and *Zea mays*, monitored the effects of the bisulfite conversion rate, the sequencing error rate, the maximum number of allowed mismatches, as well as the genome structure and size, and calculated precision, number of uniquely mapped reads, distribution of the mapped reads, run time, and memory consumption as features for benchmarking the eight read mappers mentioned above. Furthermore, we validated our findings using real-world data of *Glycine max* and showed the influence of the mapping step on DMR calling in WGBS pipelines. We found that the conversion rate had only a minor impact on the mapping quality and the number of uniquely mapped reads, whereas the error rate and the maximum number of allowed mismatches had a strong impact and leads to differences of the performance of the eight read mappers. In conclusion, we recommend BSMAP which needs the shortest run time and yields the highest precision, and Bismark which requires the smallest amount of memory and yields precision and high numbers of uniquely mapped reads.

## Introduction

It has been shown that DNA methylation is involved in several biological mechanisms and diseases such as cancer (Koch et al., 2018). Plant methylation analysis is especially interesting as 5-methyl-cytosine (5mC) is involved in the heterosis effect (Chen et al., 2018), transposon silencing, and environment-dependent transcriptome plasticity (Lauss et al., 2018). However, in addition to the complementary CG methylation being highly abundant in animals, in plants CHG and uncomplimentary CHH (H=C,T or A) methylation have evolved from the former recognition system of foreign DNA.

Whole-genome bisulfite sequencing (WGBS) is often referred to as the “gold standard” for 5mC detection because the whole genome is covered at a single-base resolution. Other methods cover only preselected genome regions enriched for cytosine-phosphate-guanine-dinucleotide (CpG) content or methylation, for example with the use of restriction enzymes in reduced representation bisulfite sequencing (rrBS) (Sun et al., 2015), or methylated DNA immune precipitation, followed by next generation sequencing (MeDIP-seq) (Bock et al., 2010; Aberg et al., 2017).

**Figure 1:**
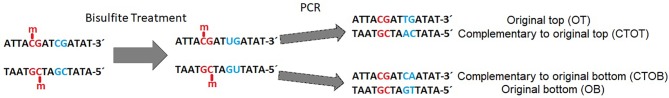
Example of a DNA double strand with methylated (red) and unmethylated (blue) CpG-site (cytosine-phosphate-guanine-dinucleotide) before and after bisulfite treatment and polymerase chain reaction (PCR). Methylated cytosine is not affected by bisulfite, whereas unmethylated cytosine is converted to uracil and further on to thymine during PCR in the original top strand, and to adenine in the complementary top strand [adapted from (Grehl et al., 2018)].

Bisulfite-mediated conversion of unmethylated cytosines to uracil, and during PCR to thymine, leads to four different strands in the data sets after sequencing: original top, complementary to original top, original bottom, and complementary to original bottom strand (**Figure 1**). Methylated cytosines remain unaffected and could be spotted by alignment of the generated sequencing reads to a reference genome or a non-bisulfite-treated control.

Critical within the bioinformatics analysis of WGBS data sets is the mapping step, as the reduced alphabet leads to specific challenges for the mapping tools due to the bisulfite treatment (Laird, 2010).

In general, two different algorithmic approaches exist in bisulfite-read alignment tools for dealing with the unmethylated C to T conversion: the ‘wild card’ and the ‘three letter’ approach. In the wild card approach the Cs in the reference genome are replaced with the wild card ‘Y’ for pyrimidine bases and thus allows for the alignment of Cs (methylated Cs) and Ts (possibly unmethylated Cs). The alignment itself is based on matching seeds (k-mers) to the reference and then extending them. In the three letter approach the alphabet of the genome and the reads is reduced to {A, G, T}, by converting all Cs in the reference sequence and in the read data to Ts. Afterwards, the reads are mapped by conventional mappers such as Bowtie, Bowtie2, or bwa, so the alignment of bisulfite data profits directly from the improvements of traditional mappers.

One study already focused on the benchmarking of rrBS alignment using simulated rrBS and real human lung tissue data sets (Sun et al., 2018). Kunde-Ramamoorthy et al. (2014) evaluated the mapping performance of five mapping tools in WGBS datasets of human peripheral blood lymphocyte and a hair follicle. Another study has been performed in plants, showing that the tool BismarkBwt2 performed best in terms of sensitivity and precision, not accounting for the coverage distribution across the reference genome (Omony et al., 2019). In contrast, our study is focused on simulated WGBS in plants, covering different species with a different amount of repetitive sequences. In addition, little is known about the mapping behavior in crop plants. Furthermore, as all former studies did not systematically account for different parameter settings such as the number of mismatches, we evaluated this parameter in more detail.

There is need for an extensive qualitative and quantitative benchmarking of alignment tools, to avoid the false interpretation of results in DNA methylation studies and to enable the application of precise, efficient, and user-friendly pipelines. The known “truth” is especially important, and this could be generated by benchmarking datasets of simulated and, thus, known read data, to calculate the quality of scores in multiclass hypothesis testing. In terms of quantitative comparison, time efficiency, amount of uniquely mapped reads, and consumption of RAM has to be monitored, as well as the overall distribution of mapped reads, to look for genomic regions with systematically lower coverages.

## Material and Methods

*Arabidopsis thaliana*, *Brassica napus*, *Glycine max*, *Solanum tuberosum*, and *Zea mays* (**Table 1**) have been examined to reveal potential inter-species variability in terms of mappability. These species have been chosen to cover different agronomically relevant plant families, different genome sizes, and different assembly qualities. All reference genomes were downloaded from http://plants.ensembl.org. We simulated WGBS datasets for 2 x 150 base pair paired-end reads for the five different plant genomes, using the open-source WGBS simulation tool: Sherman (https://www.bioinformatics.babraham.ac.uk/projects/sherman/), which has been developed at the Babraham Institute. The reads have been simulated. We chose 150bp paired-end sequencing for our benchmarking study as it is the mostly applied and proposed sequencing option for WGBS experiments. In doing so, small repeats below the total fragment size of 500-700bp could be covered, which is especially important for repeat-rich (crop) plants. Furthermore, choosing a parameter set of 150bp paired end, facilitates the necessary multiplexing with non-bisulfite libraries during sequencing.

**Table 1:** Five species included in this benchmarking study with the size of the reference genome and the used reference genome version which has been taken for the simulation of the read datasets.

Species	*Arabidopsis thaliana*	*Brassica napus*	*Glycine max*	*Solanum tuberosum*	*Zea mays*
**Genome Size (bp)**	135,670,229	738,357,821	955,377,461	727,424,546	2,104,350,183
**Genome version**	TAIR10	AST_PRJEB5043_v1	Glycine_max_v2.1	SolTub_3.0	B73 RefGen_v4
**Repeats in %**	<23 (Flutre et al., 2011)	~48 (Liu et al., 2018)	~57 (Schmutz et al., 2010)	~49 (Mehra et al., 2015)	~75 (Wolf et al., 2015)
**Citation**	Lamesch et al., 2012	Chalhoub et al., 2014	Schmutz et al., 2010	Xu et al., 2011	Schnable et al., 2009

The proportion of repetitive sequences is given as the estimated value over the genome.

**Figure 2:**
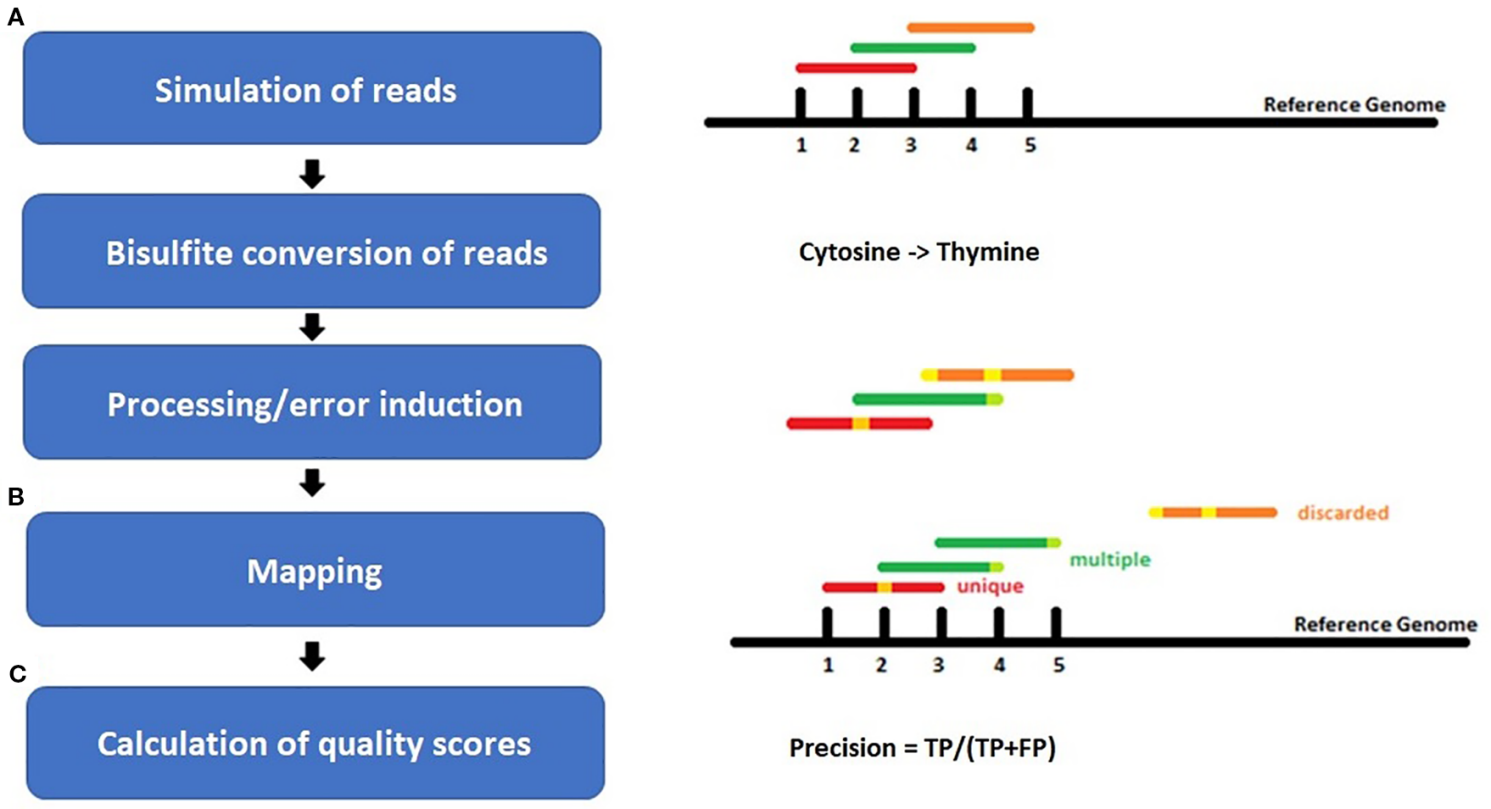
Workflow of the experiment setup for **(A)** simulation of bisulfite-treated reads based on a reference genome using the tool Sherman, including bisulfite conversion and error induction. Afterwards **(B)** mapping of the simulated datasets and **(C)** calculation of quality scores. The color coding shows the different classes of reads after mapping: red = uniquely mapped, green multiply mapped, orange= discarded/unmapped reads.

For each species, benchmarking datasets in 5-fold sequencing depth, three bisulfite conversion rates [90%, 98%, 100%], and four different sequencing error rates [0%, 0.1%, 0.5%, 1%] were simulated. The sequencing errors were modelled to account for more likely errors at the end of a read, like in real world sequencing data (**Figure 2**). Whereas the overall resulting phred score of 30 is equivalent to an error rate of 0.1% or 1 in 1000 wrong base calls. Illumina HiSeq sequencing yields an error rate of 0.0034-1% while PacBio shows 5–10% false base calls (Escalona et al., 2016). We decided to include a 98% conversion rate as this is usually guaranteed by sequencing facilities, and 90% to look for a value below this threshold.

For mapping the simulated WGBS reads to the genomes, we tested several wild-card and three-letter mappers: Bismark (Krueger and Andrews, 2011), BSMAP (Xi and Li, 2009), BS-Seeker2 (Guo et al., 2013), Bwa-meth (Pedersen et al., 2014), GEM3 (Marco-Sola et al., 2012), GSNAP (Wu and Nacu, 2010), and Segemehl (Hoffmann et al., 2009; Otto et al., 2012). These mappers differ in terms of their “age”, number of citations, and indexing strategy (**Table 2**). For further insight into mapping and indexing strategies, as well as for an insight into the underlying algorithmic approaches we recommend (Tran et al., 2014).

**Table 2:** Bisulfite Read mapping tools evaluated in this survey, listed by their mapping and indexing strategy.

Mapper name	Strategy	Indexing	Version	Citations
**Bismark**	3 letter	BWT (bowtie 2/bowtie 1)	0.19.1	1.176
**BSMAP**	wild-card	Hash table (SOAP)	2.73	3
**BS-Seeker2**	3 letter	BWT (bowtie 2)	2.1.5	135
**Bwa-meth**	3 letter	BWA mem	0.2.2	3
**GEM3**	3 letter	Custom FM index	3.6.1-2	236
**GSNAP**	wild-card	Hash table	2019-06-10	83
**Segemehl**	wild-card	Enhanced suffix array	0.2.0	283

The number of citations is based on the Web of Science Core Collection (date: 19.1.2020).

Bismark (Krueger and Andrews, 2011), one of the most cited three letter mapper for bisulfite-sequencing data, first converts the reads and the genome into two versions: a C-to-T and a G-to-A version. Afterwards, the two read versions are aligned to the two versions of the reference genome with the goal of detecting to which of the four strands (**Figure 1**) the read fits. This alignment is performed by four parallel instances of either Bowtie (Langmead et al., 2009), one of the fastest mappers for NGS data, or Bowtie2 (Langmead and Salzberg, 2012), an improved version of bowtie, that allows gapped alignment.

BSMAP (Xi and Li, 2009) is included in the list for being the first mapper for the alignment of bisulfite data. It uses an efficient HASH table the seeding algorithm for indexing the genome, bitwise masking each nucleotide in the reads and the reference and matching them to each other in an efficient way.

GSNAP (Wu and Nacu, 2010) is a general purpose mapper that can also deal with bisulfite data. Like BSMAP, it is based on special hash tables and uses a wild-card approach to match read seeds to genome regions. Since its original publication several improvements of the algorithms have been made by increasing the length of the hashed k-mers, adding a compression mechanism and using enhanced suffix arrays (Wu et al., 2016).

BS-Seeker2 (Guo et al., 2013) is the extension of BS Seeker (Chen et al., 2010) for mapping bisulfite data and deploys a three letter approach. In addition to performing a gapped alignment it can filter out reads with incomplete bisulfite conversion, in this way increasing the specificity.

Compared to the other tools in this benchmark Bwa-meth (Pedersen et al., 2014) is a relatively new mapper for bisulfite data. It is based on BWA-mem aligner (Li and Durbin, 2009; Li and Durbin, 2010) and it is advertised as a fast and accurate aligner.

Segemehl was originally designed as a general purpose mapper (Hoffmann et al., 2009) but has been extended to handle bisulfite data (Otto et al., 2012). Segemehl achieves a high sensitivity by using a wild-card approach based on enhanced suffix arrays for the seed search and the Myers bit-vector algorithm for computing semi-global alignments.

The eight mappers were used to map the simulated reads with 0, 1, 2, and 3 mismatches allowed in the read. As Bowtie2 and Bwa-meth do not allow setting the total number of mismatches in a read as a parameter, but in the seed instead, we performed our analysis on the basis of 0 mismatches in the seed for these mapping tools. Other parameter settings, such as the number of threads used, were the default values of the mentioned tools if not stated otherwise and are comparable across the different tools. All scripts are available at git-hub (https://github.com/grehl/benchWGBSmap).

After mapping, the reads can be classified into three different classes: i) discarded reads that could not be mapped, ii) multiple mapped reads that could be aligned but to more than one position on the reference genome because of sequence similarities, and iii) uniquely mapped reads, which have been mapped to one position only.

For further evaluation, we used only uniquely mapped reads. Since we did not account for insertions and deletions, we have considered only the first base of the read at its genome position. When calculating the quality scores, we have compared the true and the predicted position of a read. For each read the true genome origin is known, since Sherman encodes it in the read name, while the predicted position is derived from the alignment files.

The quality scores computed are the amount of unique reads considered, the precision, the memory consumption, and the time consumption of the tools. Furthermore, we looked at the read distribution over the reference genome to account for systematic mapping deficiencies.

The precision of a mapping tool for a data set has been computed using the formula for macro-averaged precision (macroAvgPrecision)(TP = true positives, FP = false positives):

$$macroAvgPrecision\;=\frac{\sum_{i=1}^{N}\left(\frac{TP_{i}}{TP_{i}+FP_{i}}\right)}{N}$$

We first calculated the precision for every position i, summed over all positions and divided by the total number of positions N. The macro-averaging was chosen as it weights FP higher than in the micro-averaging calculation of the precision. We furthermore used “precision” in this manuscript instead of “macro-averaged precision”.

**Figure 3:**
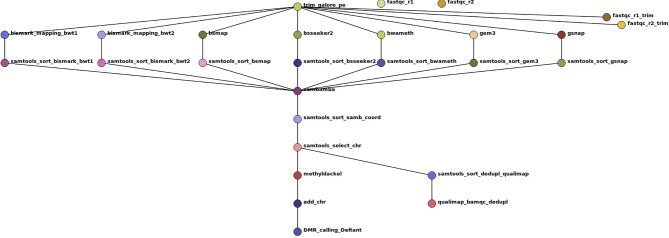
Workflow show as Directed Acyclic Graph (DAG) of the benchmark experiments using 7 mapping tools on a real-world dataset of *Glycine max* root hair grown under two different temperatures to exemplarily compare the mapping performance and the influence of the mapping tools on DMR calling. The workflow includes two quality checks on the raw reads prior and after trimming with trim galore using fastqc, the mapping, a samtools sorting step to account for different output formats of the tools, a sambamba deduplication step, coordinate sorting, exclusion of scaffold mappings, methylation level calling using methyldackel and after adding “chr” to the methylation report output the final DMR calling with Defiant. To evaluate the bam quality we used the qualimap bamqc function.

To evaluate the impact of the tested tools on DMR calling and to show the reliability of our simulated benchmark study, we included a real-world dataset of *Glycine max* root hair samples grown under 25°C and 40°C (Hossain et al., 2017). For automatization we implemented a snakemake pipeline (Köster and Rahmann, 2012), shown in **Figure 3**. Other tools used in this pipeline are: trim galore (http://www.bioinformatics.babraham.ac.uk/projects/trim_galore/), fastqc (Leggett et al., 2013), qualimap (García-Alcalde et al., 2012), samtools (Li et al., 2009), sambamba (Tarasov et al., 2015), methyldackel (https://github.com/dpryan79/MethylDackel) Defiant (v.1.1.6) (Condon et al., 2018) [parameter settings: -c 10 -v ‘BY’ -CpN 5 -p 0.05 -P 10], and Circos (Krzywinski et al., 2009). The mapping parameter sets were comparable and allowed 0 mismatches. As Segemehl showed an extensive memory consumption and runtime we had to exclude this mapper for the qualitative benchmark study and the mapping of the real dataset. For the DMR calling we had to exclude BS-Seeker2, as the flag information did not follow standard formats, so the files could not reliably be used for methylation calling. The settings for DMR calling were: minimum 10-fold coverage, minimum 5 CpN in one DMR, and minimum +/-10% methylation difference between the two treatments with a maximum p-value of 0.05. Analogous to the source code of the simulated benchmark study, we mainly relied on the default parameters if not stated otherwise in the script. All scripts are available online at git-hub (https://github.com/grehl/benchWGBSmap).

Simulation, mapping, and quality score calculation was performed on the IANVS High-Performance-Cluster of Martin-Luther University Halle-Wittenberg (**Table 3**). For calculation of runtime and memory consumption only one core of the login-nodes was allowed for mapping. For simplicity, to give an overview about mapping, and as the genome size is the most important factor with respect to runtime and memory consumption, we decided to focus on this factor only. A subsequent study could focus on the influence of other factors of runtime and memory consumption. The mapping of the quality benchmark was performed on the “large” and “small” nodes.

**Table 3:** IANVS Cluster Specifications.

Node type	SLURM partition*	Qty.	CPU	Cores (total)	SMT threads (total)	Clock speed (GHz)	RAM (GiB)	Storage	InfiniBand blocking factor**	Remarks
login	–	2	2x12-core Intel Xeon E5-2680v3	24	48	2.50	256	GPFS over IB	1:8	–
small	standard	180					128		1:2 or 1:8	hostnames: small[001-180]
large		76					256			hostnames: large[001-076]
gpu	gpu	12								hostnames: gpu[01-12]
special	Special	2	4x 10-core E5-4620v3	40	80	2.00	1024		1:8	hostnames: special001, special 002

*a “partition” in SLURM terms means “a group of machines to which one may submit cluster jobs”.

**Nodes on the same InfiniBand switch always have their full bandwidth available when communicating with each other. However, if nodes want to communicate over switch boundaries, their available bandwidth might be reduced due to contention on the switch. The “blocking factor” is the maximum reduction of bandwidth that can occur in a case like this.

## Results

**Figure 4:**
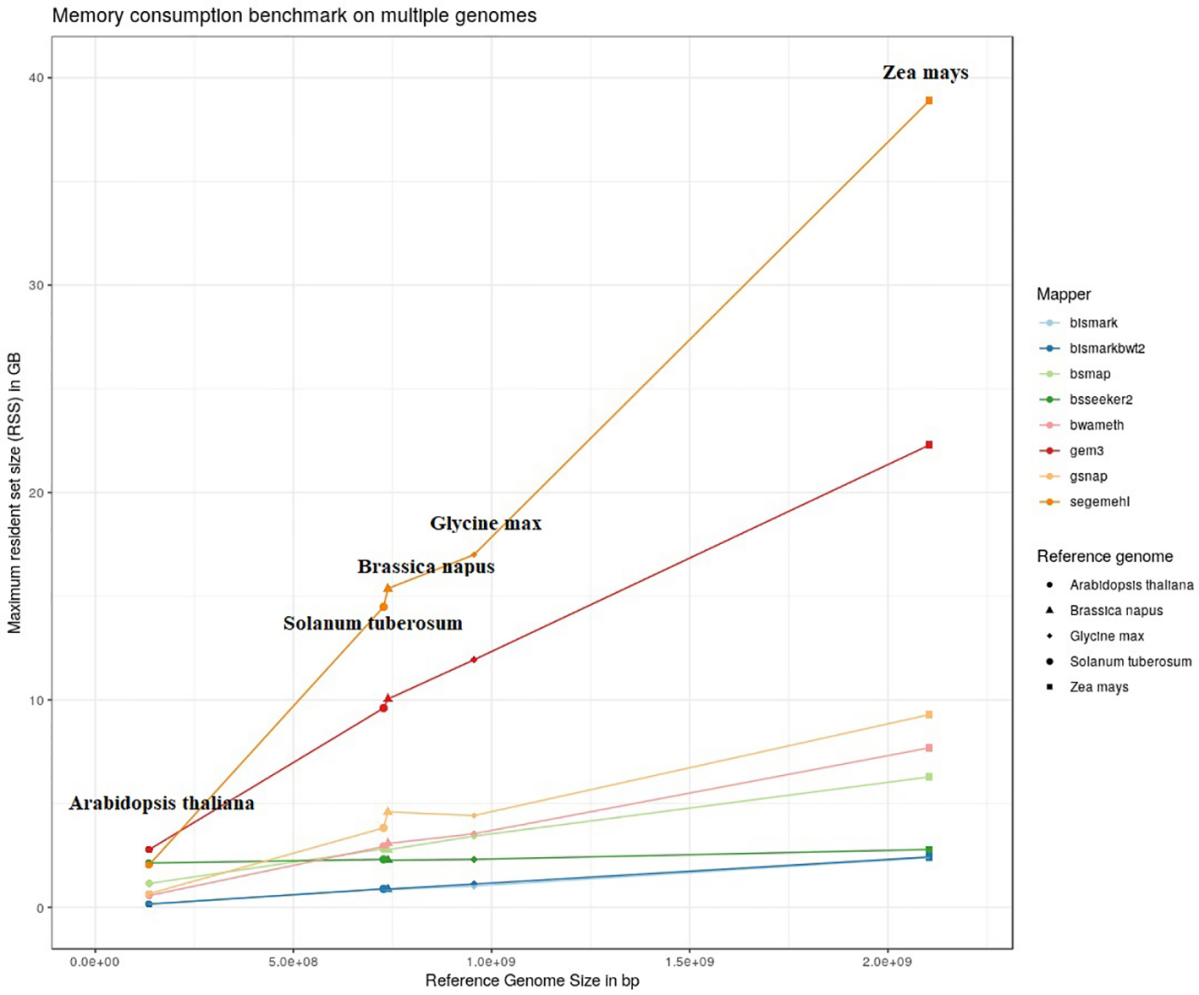
Maximum resident set size in GB of 8 mappers for 5fold simulated bisulfite converted datasets out of five reference genomes (*Arabidopsis thaliana, Brassica napus, Glycine max, Solanum tuberosum, Zea mays*). 0 % error rate, 100% conversion rate, 0 mismatch allowed.

**Figure 5:**
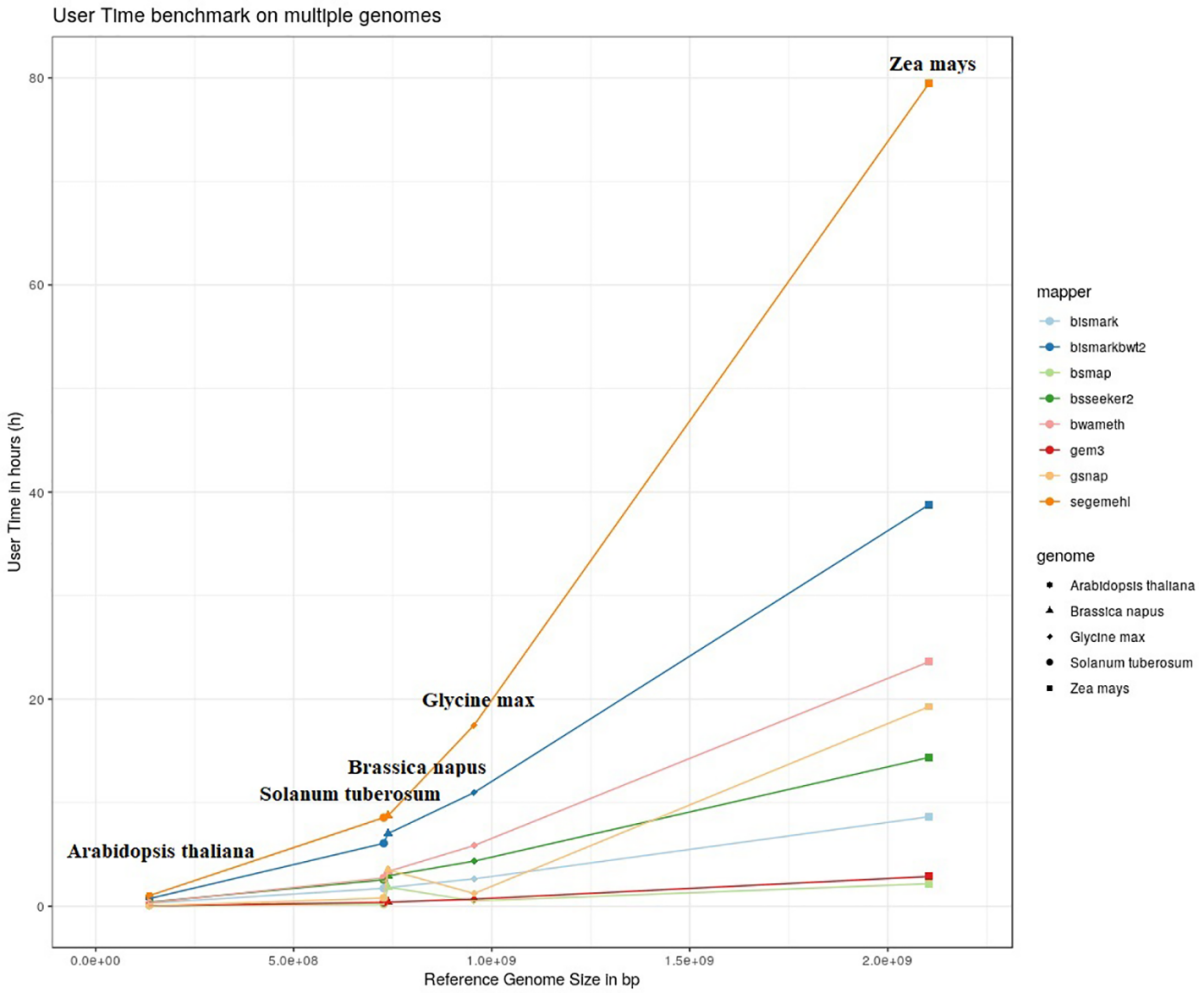
User timer in hours of 8 mappers for 5fold simulated bisulfite converted datasets out of five reference genomes (*Arabidopsis thaliana, Brassica napus, Glycine max, Solanum tuberosum, Zea mays*). 0 % error rate, 100% conversion rate, 0 mismatch allowed.

The results of our quantitative benchmark studies for memory consumption (**Figure 4**) and runtime (**Figure 5**) are shown for the eight mappers in relation to the size of the reference genome. The memory consumption ranged from 0.1 GB for the mapping of the *Arabidopsis thaliana* dataset with Bismark, either using bowtie or bowtie2, to 39 GB for the mapping of the *Zea mays* dataset with Segemehl. All datasets had a 100% conversion rate, a 0% error rate and, 0 mismatches were allowed during the mapping. Similar patterns in the memory consumption and runtime have also been observed for datasets with other parameter settings. In terms of runtime, the user time is depicted, ranging from a few minutes for all mappers using the *Arabidopsis thaliana* dataset, to 79 h for the mapping of a *Zea mays* dataset with Segemehl. Overall BSMAP took the least time, especially for large reference genomes. It is interesting to note that although *Solanum tuberosum* and *Brassica napus* have a similar genome size, and some mappers had a higher memory consumption (Segemehl, GEM3, GSNAP) and runtime (Segemehl, BismarkBwt2, GSNAP) for *Brassica napus*. This might be due to the large amount of large repetitive regions and paralogue genes within the *Brassica napus* genome, as the overall proportion of repeats is comparable to *Solanum tuberosum*.

Because of the extensive memory consumption and runtime of Segemehl, we excluded this mapper from the quality benchmark study.

**Figure 6:**
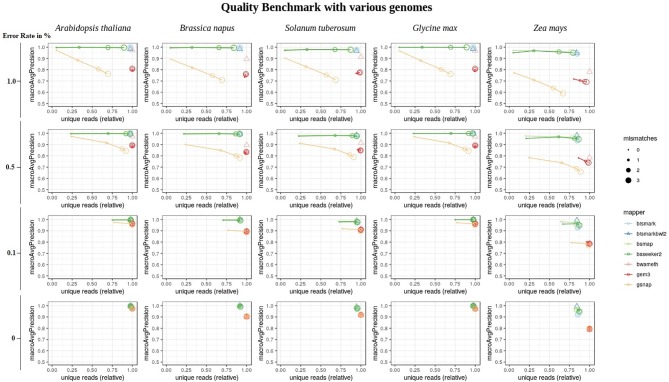
Quality benchmark of 7 mappers based on simulated bisulfite sequencing datasets in *Arabidopsis thaliana, Brassica napus, Glycine max, Solanum tuberosum,* and *Zea mays.* We simulated the datasets with 4 different error rates [0, 0.1, 0.5 and 1 %] in a 5fold coverage. For 5 out of 7 mappers we had the opportunity to allow for different numbers of mismatches [0, 1, 2, 3]. These mappers are depicted by circles. Two mappers, bismark using bowtie2 and bwameth, did not allow the adjustment for different numbers of mismatches in the entire read. They are depicted by triangles. The conversion rate had no effect and is therefore not shown in this figure. The depicted conversion rate is 100% for all data sets.

Overall, the conversion rate did not influence the number of uniquely mapped reads or the mapping quality (**Supplementary Material**). In terms of the mapping quality, in relation to the error rate, and the reference genome, we basically see three groups of mappers (**Figure 6**). The first group is independent of the allowed number of mismatches during the mapping and includes Bismark, BismarkBwt2, Bwa-meth, and GEM3. The second group consists of BSMAP and BS-Seeker2, showing an increase in the number of uniquely mapped reads with higher numbers of allowed mismatches with barely any changes in precision. The third group, including GSNAP, shows an increase in the number of uniquely mapped reads but a decrease in the precision, with a higher number of mismatches allowed. As BismarkBwt2 and bwameth do not allow setting the number of mismatches in the entire read, both are labelled with a triangle. Between the analyzed genomes we see differences for all mappers with the tendency to lower numbers of uniquely mapped reads in *Zea mays* and lower precision in *Zea mays* and *Brassica napus*.

**Figure 7:**
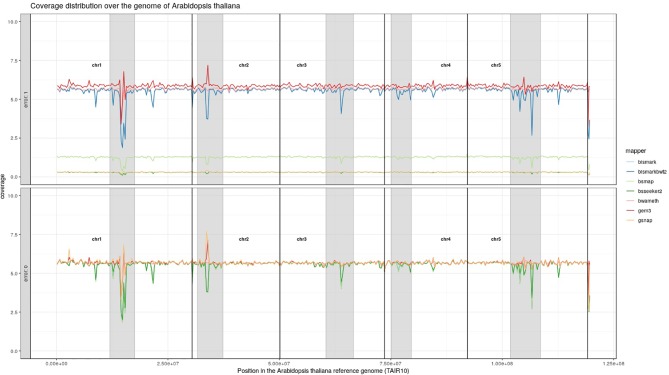
Coverage distribution over the reference genome of *Arabidopsis thaliana* (TAIR10). The lower window shows the performance of 7 mapping tools using a simulated 5fold coverage dataset with 0% induced error rate, 100% conversion rate and 0 mismatches allowed. The upper window shows a simulated 5fold coverage dataset with an induced error rate of 1%, 100% conversion rate and with 0 mismatches allowed during the mapping. The number of reads has been calculated based on the ensemblPlants “Base Pairs” information. This could cause small differences to the estimated 5fold coverage datasets. Black lines indicate the borders of chromosomes. Grey regions highlight highly repetitive regions.

**Figure 8:**
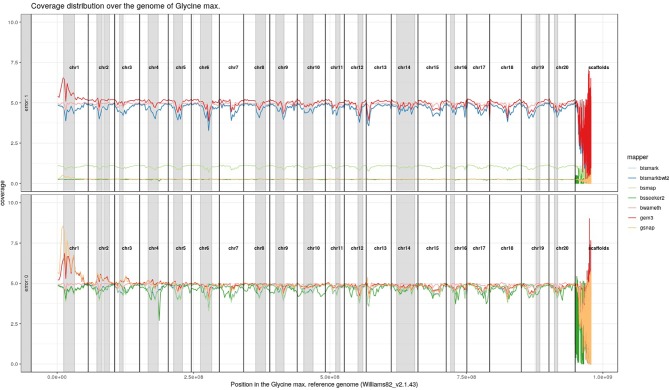
Coverage distribution over the reference genome of *Glycine max* (Williams82_v2.1.43). The lower window shows the performance of 7 mappers using a simulated 5fold coverage dataset with 0% induced error rate, 100% conversion rate, and 0 mismatches allowed. The upper window shows a simulated 5fold coverage dataset with an induced error rate of 1%, 100% conversion rate, and with 0 mismatches allowed during the mapping. Black lines indicate the borders of chromosomes. Grey regions highlight highly repetitive regions.

For *Arabidopsis thaliana* (**Figure 7**) and *Glycine max* (**Figure 8**) the distribution of reads over the reference genome is exemplarily shown for the mapping of two datasets each. The first dataset was simulated with a 100% conversion rate, a 0% error rate and was mapped with 0 mismatches allowed for all seven mapping tools, depicted in the lower window. The upper window shows a 100% conversion rate, a 1% error rate and 0 mismatches, again for all seven mapping tools. All coverage plots have a resolution of 400 windows across the whole reference genome. For higher error rates BSMAP, BS-Seeker2, and GSNAP show a severe decrease in coverage. Furthermore, we clearly see several regions with a decrease in coverage within the reference genome, independent of the error rate. In grey, we highlighted the regions which are known to contain a high percentage of repetitive sequences. Bismark and BismarkBwt2 are depicted behind each other, showing nearly the same coverage distribution. In total, Bwa-meth shows the least derivation in the coverage distribution.

**Figure 9:**
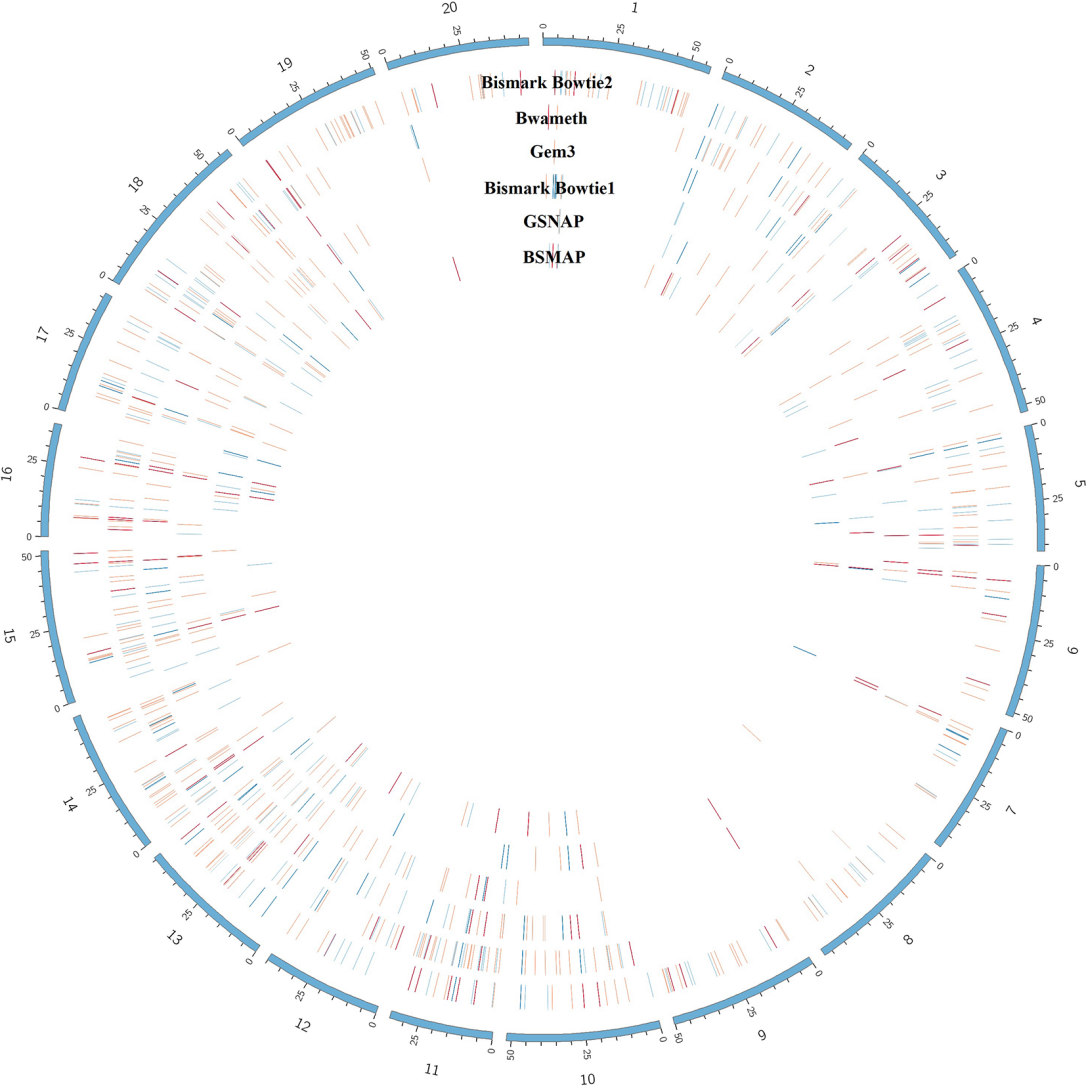
Circular plot showing the distribution of DMRs (red) as result of mapping the same dataset with 6 different mapping tools on the Glycine max_v2.1 reference genome. The outer circle shows the chromosomes of *Glycine max* in blue. Blue lines indicate hypomethylation, whereas red lines indicate hypermethylation (see the full list of DMRs at https://github.com/grehl/benchWGBSmap). Numbers of overlapping DMRs could be found in the **Supplementary Material**.

The benchmarking of the real *Glycine max* dataset resulted in proper mapped paired end read counts (**Table 4**). The last column shows the final number of DMRs. These are additionally depicted in **Figure 9**.

**Table 4:** Mean coverage of the four real data samples and the result of the DMR calling (SRR5044695 & SRR5044696 are the control and SRR5044699 & SRR5044700 are the heat stress replicates).

	SRR5044695	SRR5044696	SRR5044699	SRR5044700	DMRs
**BismarkBwt2**	15,0	14,5	17,9	19,2	281
**Bwa-meth**	32,5	29,5	35,6	38,8	256
**GEM3**	28,3	25,8	31,4	34,3	136
**BismarkBwt1**	11,3	11,0	13,9	14,7	97
**GSNAP**	10,3	9,5	12,0	12,6	70
**BSMAP**	10,5	9,9	12,2	12,7	63
**BS-Seeker2**	8,6	8,4	11,0	11,3	X

## Discussion

We performed an extensive benchmarking experiment based on simulated data to evaluate the qualitative and quantitative performance of mappers for bisulfite sequencing data in five plant species with a focus on crop plants.

In terms of user time and memory consumption, the different tools showed big differences. Especially for larger genomes. For example Segemehl used a tremendous amount of RAM and needed the most time to map the given reads onto the reference genome. For larger reference genomes (>4 GB) the genome has to be split if Segemehl needs to be used. For these two reasons, we could not use Segemehl for mapping of huge datasets such as *Zea mays*, even if it performed well in terms of precision in a pilot study. BSMAP, GEM3, and GSNAP showed only a low increase in time with the increasing size of the genome but used more memory. Bismark in particular showed a low increase for the memory consumption and a relatively low increase in run time. The large difference between Bismark and BismarkBwt2 is most likely due to the “soft clipping” function of BismarkBwt2.

The mapping quality and number of uniquely mapped reads change between the tools with *Zea mays* showing the lowest precision scores and the least number of uniquely mapped reads. This effect might be caused by the high number of repetitive sequences, which has been shown to make up to 75% of the *Zea mays* genome, containing mostly gypsy- and copia-like long, terminal repeats (LTR) (Wolf et al., 2015). For *Glycine max* the described number of repeats lays around ~57%. This also includes telomeric as well as centromeric repeats and not annotated repeats where the reference genome shows scaffolded regions (Schmutz et al., 2010). A wild-type reference genome sequencing consortium recently found 54% repeats (Xie et al., 2019). As most repeats are <50 bp (Sherman-Broyles et al., 2014), the 2 x 150 bp paired-end reads with an insert size of 200 bp – 400 bp could cover large parts of the genome uniquely. The distribution of reads across the reference genome shows a good overlap with known and long, repeat-rich regions. Some mappers such as GEM3 and GSNAP tend to map high amounts of FP in these regions. Other mappers leave these regions out, leading to a lower coverage.

In terms of precision, runtime and power to detect CpG-sites, Sun et al. (2018) found Bwa-meth and BS-Seeker2 to be the best tools based on simulated and real rrBS reads from human lung tumor tissue. However, this stands in contrast to our findings, which show precision deficiencies for Bwa-meth, with error rates above 0.1%, especially in repeat-rich and large plant genomes. BS-Seeker2 mapped reads precisely but error rates above 0.5% and with 0 mismatches allowed during mapping leads to unique mapping rates below 25%. Other studies found Bismark to yield a reasonable combination of low memory consumption, low runtime, and high quality scores (Kunde-Ramamoorthy et al., 2014; Omony et al., 2019). This could be confirmed by our study, where Bismark showed the lowest memory consumption in all tested genomes. For runtime, we see high differences between Bismark using Bowtie and BismarkBwt2 under usage of Bowtie2. The precision showed good scores for all genomes and settings, with a slight decrease for the *Zea mays* genome.

For the second part of our study we mapped the same datasets with the seven mentioned mapping tools but had to exclude BS-Seeker2 for the DMR calling. Here, we see the most unique, proper paired reads for mapping with Bwa-meth and GEM3. Surprisingly, this could not be confirmed for the DMR calling where we obtained the most DMRs using Bismark with Bowtie2 using the same parameter sets, the same tools, and the same pipeline. We can only speculate what the reason for this behavior might be. Most likely this shift in the performance difference between the tools could be caused by false positive mappings which did not heavily influence the DMR calling, as they might have been mapped to “non-sense” positions either already involved in a DMR region, not causing much harm in remote regions due to the coverage threshold of 10-fold, or, they have been evenly distributed over treatment and control datasets.

## Conclusion

In conclusion, we have shown high differences between the available mapping tools for bisulfite-treated reads based on simulated and real datasets in terms of runtime, memory consumption, and mapping quality. We see the stability of mapping quality against changes in the conversion rate, high differences between the mapping tools in terms of the number of uniquely mapped reads as well as in the capability to map correctly under the impact of higher error rates in five different genomes. Additionally, we see high differences with regard to the analyzed genome, dependent on the size and structure of repeats.

For *Arabidopsis thaliana* basically every one of the examined mapping tools could be used with a sufficient mapping rate and good quality, at least when assuming a low error rate. This holds true for low error rates in *Glycine max* mappings. For higher error rates we recommend Bwa-meth as well as Bismark, using Bowtie1 or Bowtie2. For paralogue-rich species such as *Brassica napus*, polyploid species such as *Solanum tuberosum*, or large genomes with many repetitive sequences such as in *Zea mays* we prefer correct mappings over a large number of unique mapped reads. Therefore, going with Bismark using Bowtie1 or Bowtie2 or BSMAP and BS-Seeker2 with a higher number of mismatches allowed might work well, looking at the perspective of mapping.

Altogether, we recommend BSMAP as this requires the shortest run time and yields the highest precision and Bismark which requires the smallest amount of memory and yields high precision and high numbers of uniquely mapped reads. Furthermore, Bwa-meth could be used with care in terms of precise calling of DMRs.

## Data Availability Statement

The raw data supporting the conclusions of this article will be made available by the authors, without undue reservation, to any qualified researcher.

## Author Contributions

Conceptualization: CG and MW Methodology: CG, MW Investigation: CG, MW. Writing—original draft preparation: CG and IL. Writing—review and editing: IL, BG, IG. Visualization: CG and MW. Supervision: BG, IG. Project administration: CG, BG, IG. Funding acquisition: CG, BG, IG.

## Funding

This research was funded by the state of Saxony-Anhalt and Volkswagen Stiftung. The Article Processing Charges have been supported by the open-science fund of the Martin-Luther University Halle-Wittenberg. IL was funded by the Deutsche Forschungsgemeinschaft (DFG, Germany) Research Training Group 2155 (ProMoAge).

## Conflict of Interest

The authors declare that the research was conducted in the absence of any commercial or financial relationships that could be construed as a potential conflict of interest.

## References

[B1] Aberg K. A. Chan R. F. Shabalin A. A. Zhao M. Turecki G. Staunstrup N. H. (2017). A MBD-seq protocol for large-scale methylome-wide studies with (very) low amounts of DNA. Epigenetics 12 (9), 743–750. 10.1080/15592294.2017.1335849 28703682 PMC5739096

[B2] Bock C. Tomazou E. M. Brinkman A. B. Müller F. Simmer F. Gu H. (2010). Quantitative comparison of genome-wide DNA methylation mapping technologies. Nat. Biotechnol. 28 (10), 1106–1114. 10.1038/nbt.1681 20852634 PMC3066564

[B3] Chalhoub B. Denoeud F. Liu S. Parkin I. A. Tang H. Wang X. (2014). Plant genetics. Early allopolyploid evolution in the post-Neolithic Brassica napus oilseed genome. Sci. (New York N.Y.) 345 (6199), 950–953. 10.1126/science.1253435 25146293

[B4] Chen P.-Y. Cokus S. J. Pellegrini M. (2010). BS Seeker: precise mapping for bisulfite sequencing. BMC Bioinf. 11, 203. 10.1186/1471-2105-11-203 PMC2871274 20416082

[B5] Chen X. Schönberger B. Menz J. Ludewig U. (2018). Plasticity of DNA methylation and gene expression under zinc deficiency in Arabidopsis roots. Plant Cell Physiol. 59 (9), 1790–1802 10.1093/pcp/pcy100 29800330

[B6] Condon D. E. Tran P. V. Lien Y.-C. Schug J. Georgieff M. K. Simmons R. A. (2018). Defiant: (DMRs: easy, fast, identification and ANnoTation) identifies differentially Methylated regions from iron-deficient rat hippocampus. BMC Bioinf. 19 (1), 31. 10.1186/s12859-018-2037-1 PMC5800085 29402210

[B7] Escalona M. Rocha S. Posada D. (2016). A comparison of tools for the simulation of genomic next-generation sequencing data. Nat. Rev. Genet. 17 (8), 459–469. 10.1038/nrg.2016.57 27320129 PMC5224698

[B8] Flutre T. Duprat E. Feuillet C. Quesneville H. (2011). Considering transposable element diversification in de novo annotation approaches. PloS One 6 (1), e16526. 10.1371/journal.pone.0016526 21304975 PMC3031573

[B9] García-Alcalde F. Okonechnikov K. Carbonell J. Cruz L. M. Götz S. Tarazona S. (2012). Qualimap: evaluating next-generation sequencing alignment data. Bioinf. (Oxford England) 28 (20), 2678–2679. 10.1093/bioinformatics/bts503 22914218

[B10] Grehl C. Kuhlmann M. Becker C. Glaser B. Grosse I. (2018). How to design a whole-genome Bisulfite sequencing experiment. Epigenomes 2 (4), 21. 10.3390/epigenomes2040021

[B11] Guo W. Fiziev P. Yan W. Cokus S. Sun X. Zhang M. Q. (2013). BS-Seeker2: a versatile aligning pipeline for bisulfite sequencing data. BMC Genomics 14, 774. 10.1186/1471-2164-14-774 24206606 PMC3840619

[B12] Hoffmann S. Otto C. Kurtz S. Sharma C. M. Khaitovich P. Vogel J. (2009). Fast mapping of short sequences with mismatches, insertions and deletions using index structures. PloS Comput. Biol. 5 (9), e1000502. 10.1371/journal.pcbi.1000502 19750212 PMC2730575

[B13] Hossain M. S. Kawakatsu T. Kim K. D. Zhang N. Nguyen C. T. Khan S. M. (2017). Divergent cytosine DNA methylation patterns in single-cell, soybean root hairs. New Phytol. 214 (2), 808–819. 10.1111/nph.14421 28106918

[B14] Köster J. Rahmann S. (2012). Snakemake–a scalable bioinformatics workflow engine. Bioinf. (Oxford England) 28 (19), 2520–2522. 10.1093/bioinformatics/bts480 22908215

[B15] Koch A. Joosten S. C. Feng Z. de Ruijter T. C. Draht M. X. Melotte V. (2018). Analysis of DNA methylation in cancer: location revisited. Nat. Rev. Clin. Oncol. 15 (7), 459–466. 10.1038/s41571-018-0004-4 29666440

[B16] Krueger F. Andrews S. R. (2011). Bismark: a flexible aligner and methylation caller for Bisulfite-seq applications. Bioinf. (Oxford England) 27 (11), 1571–1572. 10.1093/bioinformatics/btr167 PMC3102221 21493656

[B17] Krzywinski M. Schein J. Birol I. Connors J. Gascoyne R. Horsman D. (2009). Circos: an information aesthetic for comparative genomics. Genome Res. 19 (9), 1639–1645. 10.1101/gr.092759.109 19541911 PMC2752132

[B18] Kunde-Ramamoorthy G. Coarfa C. Laritsky E. Kessler N. J. Harris R. A. Xu M. (2014). Comparison and quantitative verification of mapping algorithms for whole-genome bisulfite sequencing. Nucleic Acids Res. 42 (6), e43. 10.1093/nar/gkt1325 24391148 PMC3973287

[B19] Laird P. W. (2010). Principles and challenges of genomewide DNA methylation analysis. Nat. Rev. Genet. 11 (3), 191–203. 10.1038/nrg2732 20125086

[B20] Lamesch P. Berardini T. Z. Li D. Swarbreck D. Wilks C. Sasidharan R. (2012). The Arabidopsis Information Resource (TAIR): improved gene annotation and new tools. Nucleic Acids Res. 40 ((Database issue)), D1202–D1210. 10.1093/nar/gkr1090 22140109 PMC3245047

[B21] Langmead B. Salzberg S. L. (2012). Fast gapped-read alignment with Bowtie 2. Nat. Methods 9 (4), 357–359. 10.1038/nmeth.1923 22388286 PMC3322381

[B22] Langmead B. Trapnell C. Pop M. Salzberg S. L. (2009). Ultrafast and memory-efficient alignment of short DNA sequences to the human genome. Genome Biol. 10 (3), R25. 10.1186/gb-2009-10-3-r25 19261174 PMC2690996

[B23] Lauss K. Wardenaar R. Oka R. van Hulten M. H. A. Guryev V. Keurentjes J. J. B. (2018). Parental DNA Methylation states are associated with heterosis in epigenetic hybrids. Plant Physiol. 176 (2), 1627–1645. 10.1104/pp.17.01054 29196538 PMC5813580

[B24] Leggett R. M. Ramirez-Gonzalez R. H. Clavijo B. J. Waite D. Davey R. P. (2013). Sequencing quality assessment tools to enable data-driven informatics for high throughput genomics. Front. In Genet. 4, 288. 10.3389/fgene.2013.00288 24381581 PMC3865868

[B25] Li H. Durbin R. (2009). Fast and accurate short read alignment with Burrows-Wheeler transform. Bioinf. (Oxford England) 25 (14), 1754–1760. 10.1093/bioinformatics/btp324 PMC2705234 19451168

[B26] Li H. Durbin R. (2010). Fast and accurate long-read alignment with Burrows-Wheeler transform. Bioinf. (Oxford England) 26 (5), 589–595. 10.1093/bioinformatics/btp698 PMC2828108 20080505

[B27] Li H. Handsaker B. Wysoker A. Fennell T. Ruan J. Homer N. (2009). The sequence alignment/map format and SAMtools. Bioinf. (Oxford England) 25 (16), 2078–2079. 10.1093/bioinformatics/btp352 PMC2723002 19505943

[B28] Liu S. Snowdon R. Chalhoub B. (Eds.) (2018). The Brassica napus Genome (Cham: Springer International Publishing (Compendium of Plant Genomes)). 10.1007/978-3-319-43694-4

[B29] Marco-Sola S. Sammeth M. Guigó R. Ribeca P. (2012). The GEM mapper: fast, accurate and versatile alignment by filtration. Nat. Methods 9 (12), 1185–1188. 10.1038/nmeth.2221 23103880

[B30] Mehra M. Gangwar I. Shankar R. Houben A. (2015). A deluge of complex repeats. The solanum genome. PloS One 10 (8), e0133962. 10.1371/journal.pone.0133962 26241045 PMC4524691

[B31] Omony J. Nussbaumer T. Gutzat R. (2019). DNA methylation analysis in plants. Review of computational tools and future perspectives. Briefings In Bioinf. 38 (5), 285. 10.1093/bib/bbz039 PMC7612617 31220217

[B32] Otto C. Stadler P. F. Hoffmann S. (2012). Fast and sensitive mapping of bisulfite-treated sequencing data. Bioinf. (Oxford England) 28 (13), 1698–1704. 10.1093/bioinformatics/bts254 22581174

[B33] Pedersen B. Eyring K. De S. Yang I. Schwartz D. (2014). Fast and accurate alignment of long bisulfite-seq reads. Prepring: arXiv:1401.1129v2. Bioinf. (Oxford England). 5/13/2014.

[B34] Schmutz J. Cannon S. B. Schlueter J. Ma J. Mitros T. Nelson W. (2010). Genome sequence of the palaeopolyploid soybean. Nature 463 (7278), 178–183. 10.1038/nature08670 20075913

[B35] Schnable P. S. Ware D. Fulton R. S. Stein J. C. Wei F. Pasternak S. (2009). The B73 maize genome: complexity, diversity, and dynamics. Sci. (New York N.Y.) 326 (5956), 1112–1115. 10.1126/science.1178534 19965430

[B36] Sherman-Broyles S. Bombarely A. Grimwood J. Schmutz J. Doyle J. (2014). Complete plastome sequences from Glycine syndetika and six additional perennial wild relatives of soybean. G3 (Bethesda Md.) 4 (10), 2023–2033. 10.1534/g3.114.012690 PMC4199708 25155272

[B37] Sun Z. Cunningham J. Slager S. Kocher J.-P. (2015). Base resolution methylome profiling: considerations in platform selection, data preprocessing and analysis. Epigenomics 7 (5), 813–828. 10.2217/epi.15.21 26366945 PMC4790440

[B38] Sun X. Han Y. Zhou L. Chen E. Lu B. Liu Y. (2018). A comprehensive evaluation of alignment software for reduced representation bisulfite sequencing data. Bioinf. (Oxford England) 34 (16), 2715–2723. 10.1093/bioinformatics/bty174 29579198

[B39] Tarasov A. Vilella A. J. Cuppen E. Nijman I. J. Prins P. (2015). Sambamba: fast processing of NGS alignment formats. Bioinf. (Oxford England) 31 (12), 2032–2034. 10.1093/bioinformatics/btv098 PMC4765878 25697820

[B40] Tran H. Porter J. Sun M.-A. Xie H. Zhang L. (2014). Objective and comprehensive evaluation of bisulfite short read mapping tools. Adv. In Bioinf. 2014, 472045. 10.1155/2014/472045 PMC4009243 24839440

[B41] Wolf P. G. Sessa E. B. Marchant D. B. Li F.-W. Rothfels C. J. Sigel E. M. (2015). An exploration into fern genome space. Genome Biol. Evol. 7 (9), 2533–2544. 10.1093/gbe/evv163 26311176 PMC4607520

[B42] Wu T. D. Nacu S. (2010). Fast and SNP-tolerant detection of complex variants and splicing in short reads. Bioinf. (Oxford England) 26 (7), 873–881. 10.1093/bioinformatics/btq057 PMC2844994 20147302

[B43] Wu T. D. Reeder J. Lawrence M. Becker G. Brauer M. J. (2016). GMAP and GSNAP for genomic sequence alignment: enhancements to speed, accuracy, and functionality. Methods In Mol. Biol. (Clifton N.J.) 1418, 283–334. 10.1007/978-1-4939-3578-9_15 27008021

[B44] Xi Y. Li W. (2009). BSMAP: whole genome bisulfite sequence MAPping program. BMC Bioinf. 10, 232. 10.1186/1471-2105-10-232 PMC2724425 19635165

[B45] Xie M. Chung C. Y.-L. Li M.-W. Wong F.-L. Wang X. Liu A. (2019). A reference-grade wild soybean genome. Nat. Commun. 10 (1), 1216. 10.1038/s41467-019-09142-9 30872580 PMC6418295

[B46] Xu X. Pan S. Cheng S. Zhang B. Mu D. Ni P. (2011). Genome sequence and analysis of the tuber crop potato. Nature 475 (7355), 189–195. 10.1038/nature10158 21743474

