# Supplementary material for: Performance of Mapping Approaches for Whole-Genome Bisulfite Sequencing Data in Crop Plants
Source: Front Plant Sci. 2020 Feb 28;11:176. doi: 10.3389/fpls.2020.00176 (PMC7093021; doi:10.3389/fpls.2020.00176)
Supplement: Supplementary file 4 [file Table_4.docx]

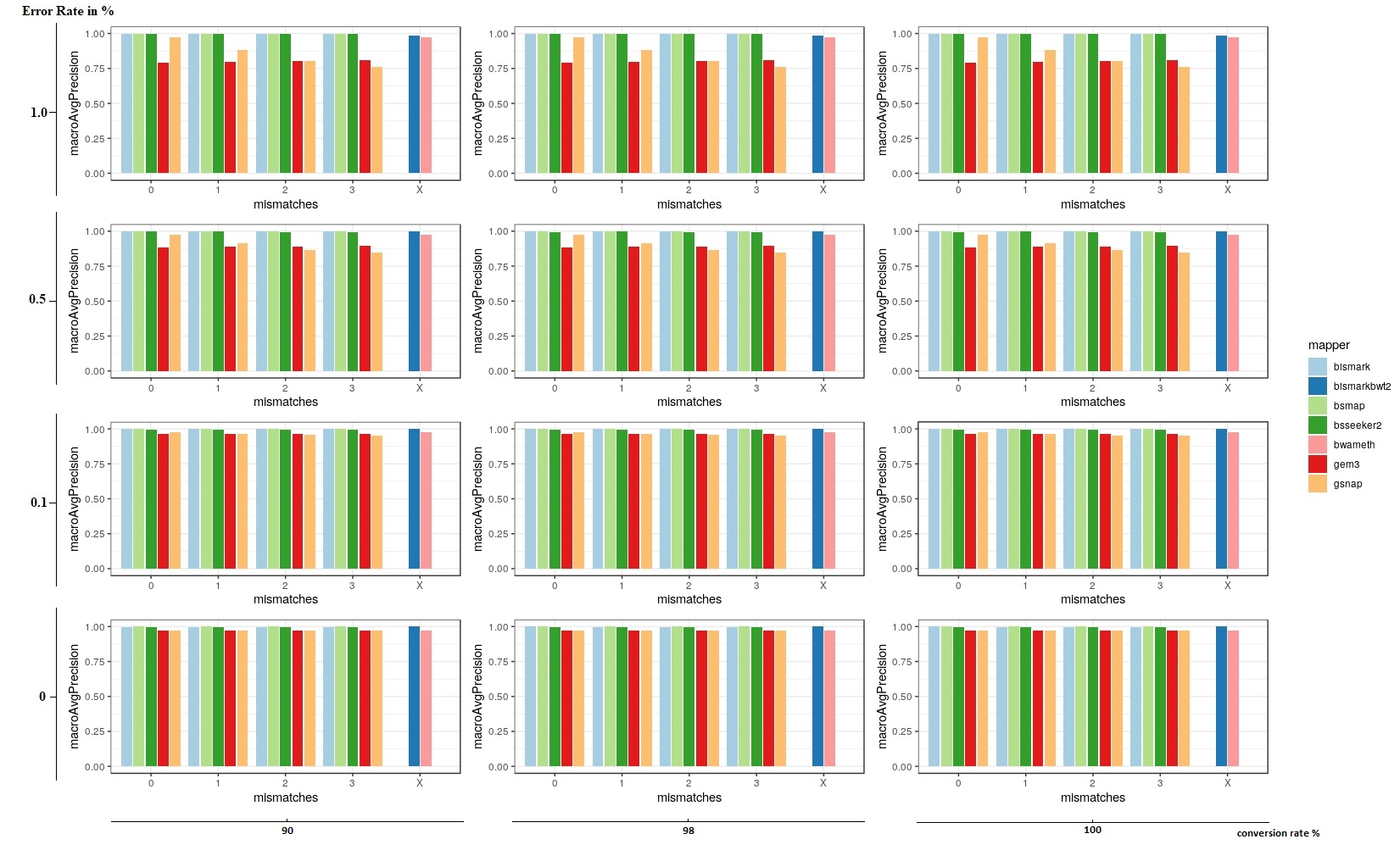


Figure 1: macroAvgPrecision of 7 mappers based on simulated bisulfite sequencing datasets in Arabidopsis thaliana. We simulated the datasets with 4 different error rates [0, 0.1, 0.5 and 1 %] and three different conversion rates [90, 98 and 100%] in a 5fold coverage. For 5 out of 7 mappers we had the opportunity to allow for different numbers of mismatches [0, 1, 2, 3]. Two mappers, bismark using bowtie2 and bwameth, did not allow the adjustment for different numbers of mismatches in the entire read. They are depicted by [X] on the y-axis.


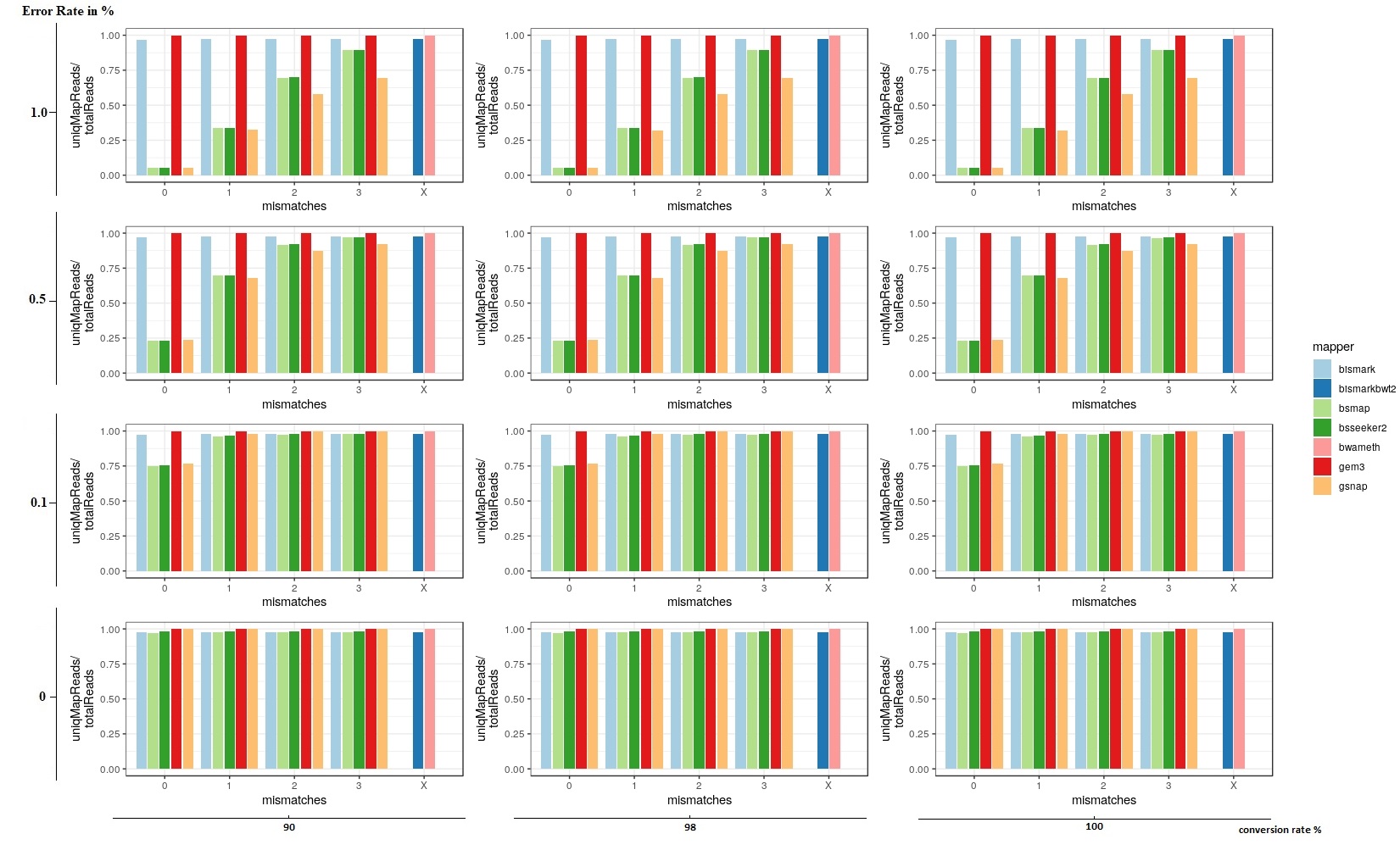


Figure 2: Relative amount of uniquely mapped reads of 7 mappers based on simulated bisulfite sequencing datasets in Arabidopsis thaliana. We simulated the datasets with 4 different error rates [0, 0.1, 0.5 and 1 %] and three different conversion rates [90, 98 and 100%] in a 5fold coverage. For 5 out of 7 mappers we had the opportunity to allow for different numbers of mismatches [0, 1, 2, 3]. Two mappers, bismark using bowtie2 and bwameth, did not allow the adjustment for different numbers of mismatches in the entire read. They are depicted by [X] on the y-axis.


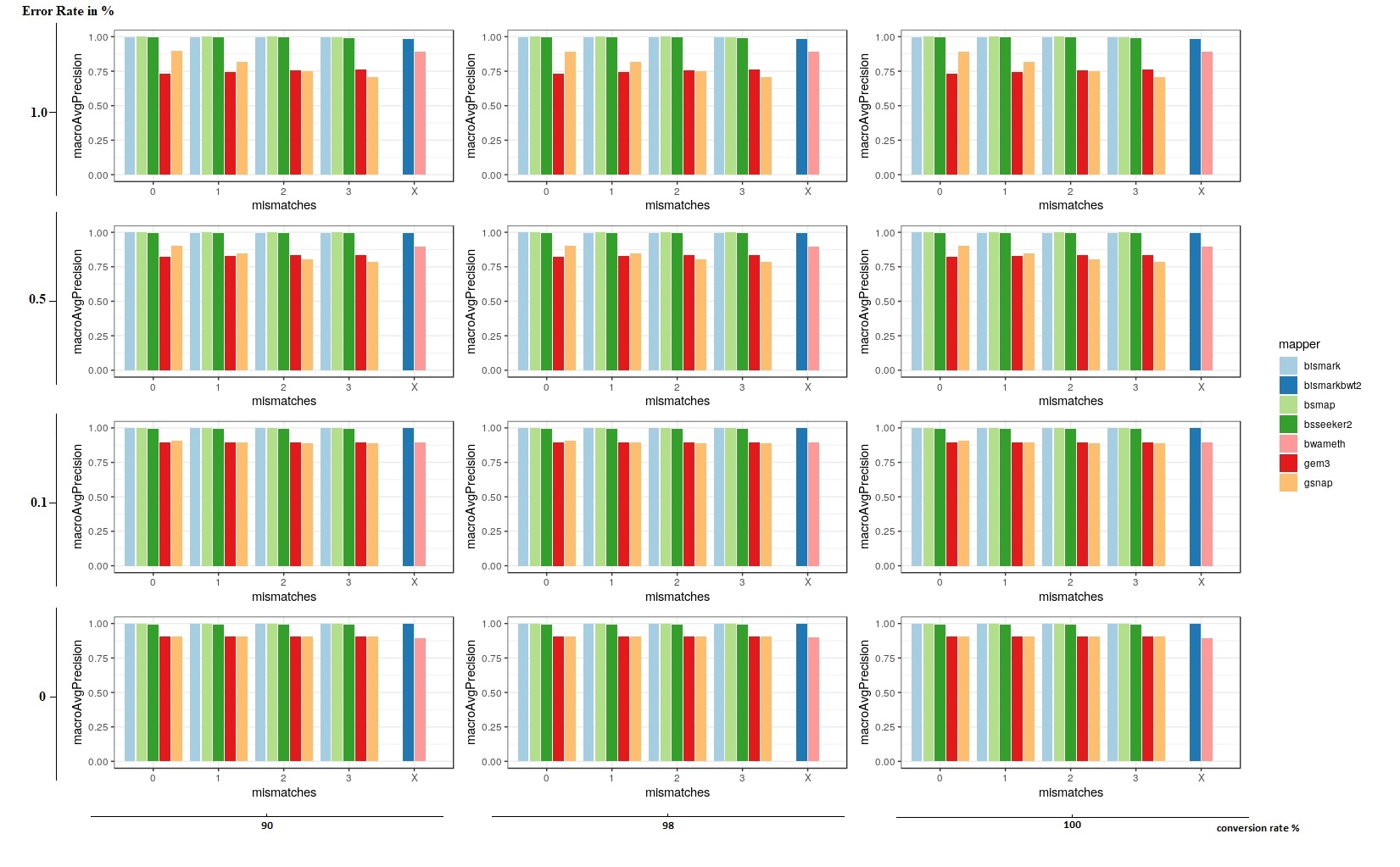


Figure 3: macroAvgPrecision of 7 mappers based on simulated bisulfite sequencing datasets in Brassica napus. We simulated the datasets with 4 different error rates [0, 0.1, 0.5 and 1 %] and three different conversion rates [90, 98 and 100%] in a 5fold coverage. For 5 out of 7 mappers we had the opportunity to allow for different numbers of mismatches [0, 1, 2, 3]. Two mappers, bismark using bowtie2 and bwameth, did not allow the adjustment for different numbers of mismatches in the entire read. They are depicted by [X] on the y-axis.


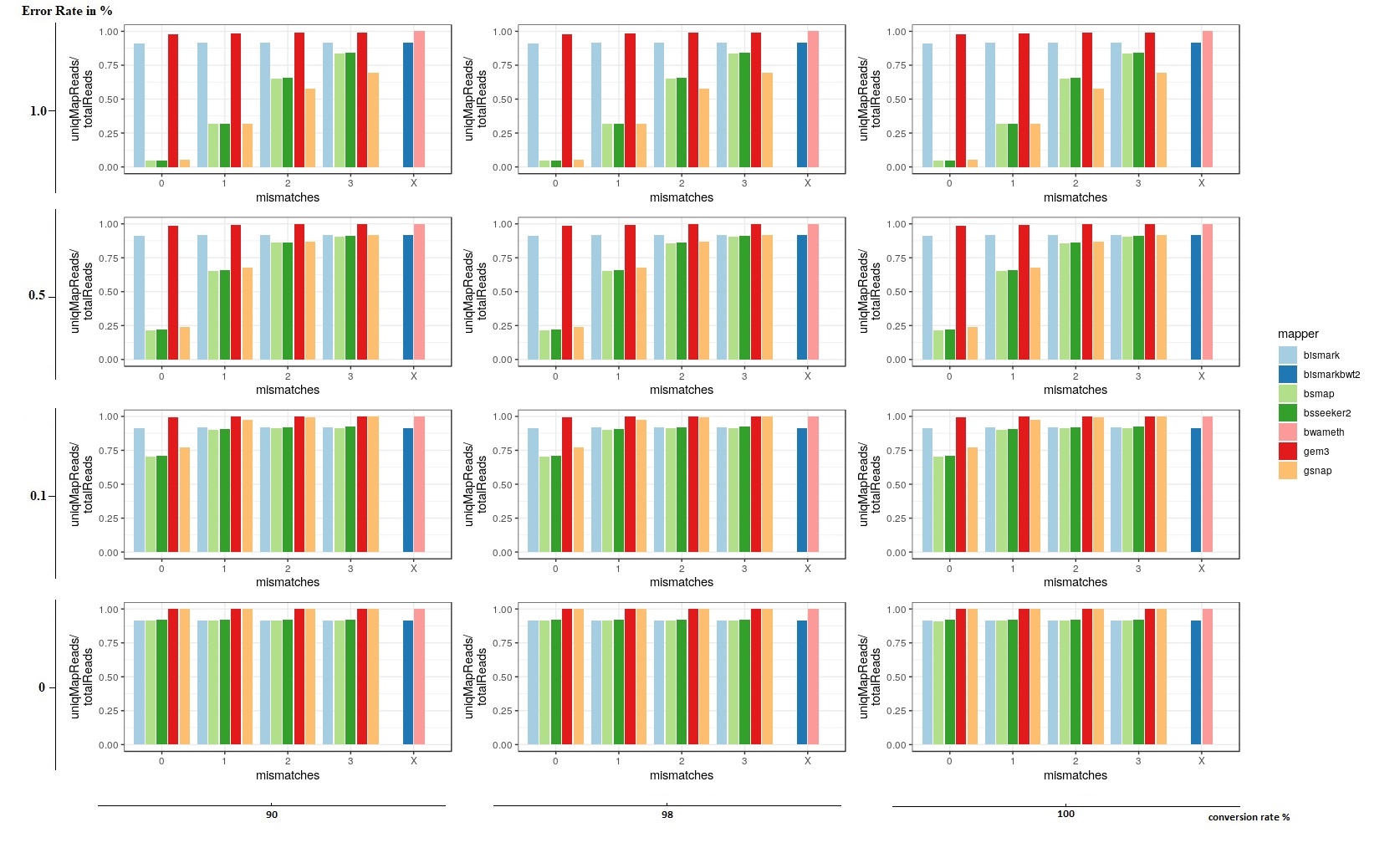


Figure 4: Relative amount of uniquely mapped reads of 7 mappers based on simulated bisulfite sequencing datasets in Brassica napus. We simulated the datasets with 4 different error rates [0, 0.1, 0.5 and 1 %] and three different conversion rates [90, 98 and 100%] in a 5fold coverage. For 5 out of 7 mappers we had the opportunity to allow for different numbers of mismatches [0, 1, 2, 3]. Two mappers, bismark using bowtie2 and bwameth, did not allow the adjustment for different numbers of mismatches in the entire read. They are depicted by [X] on the y-axis.


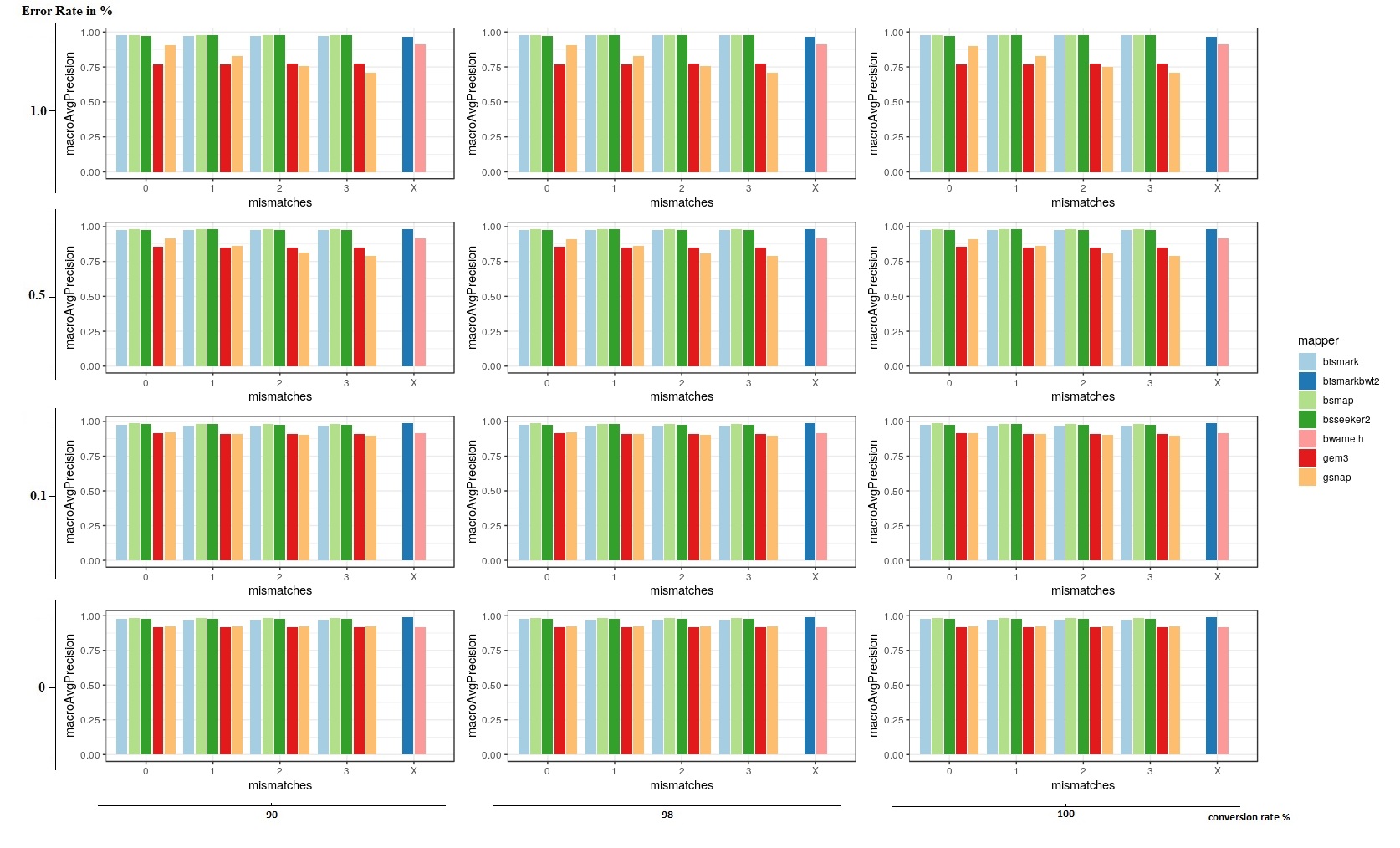


Figure 5: macroAvgPrecision of 7 mappers based on simulated bisulfite sequencing datasets in Glycine max. We simulated the datasets with 4 different error rates [0, 0.1, 0.5 and 1 %] and three different conversion rates [90, 98 and 100%] in a 5fold coverage. For 5 out of 7 mappers we had the opportunity to allow for different numbers of mismatches [0, 1, 2, 3]. Two mappers, bismark using bowtie2 and bwameth, did not allow the adjustment for different numbers of mismatches in the entire read. They are depicted by [X] on the y-axis.


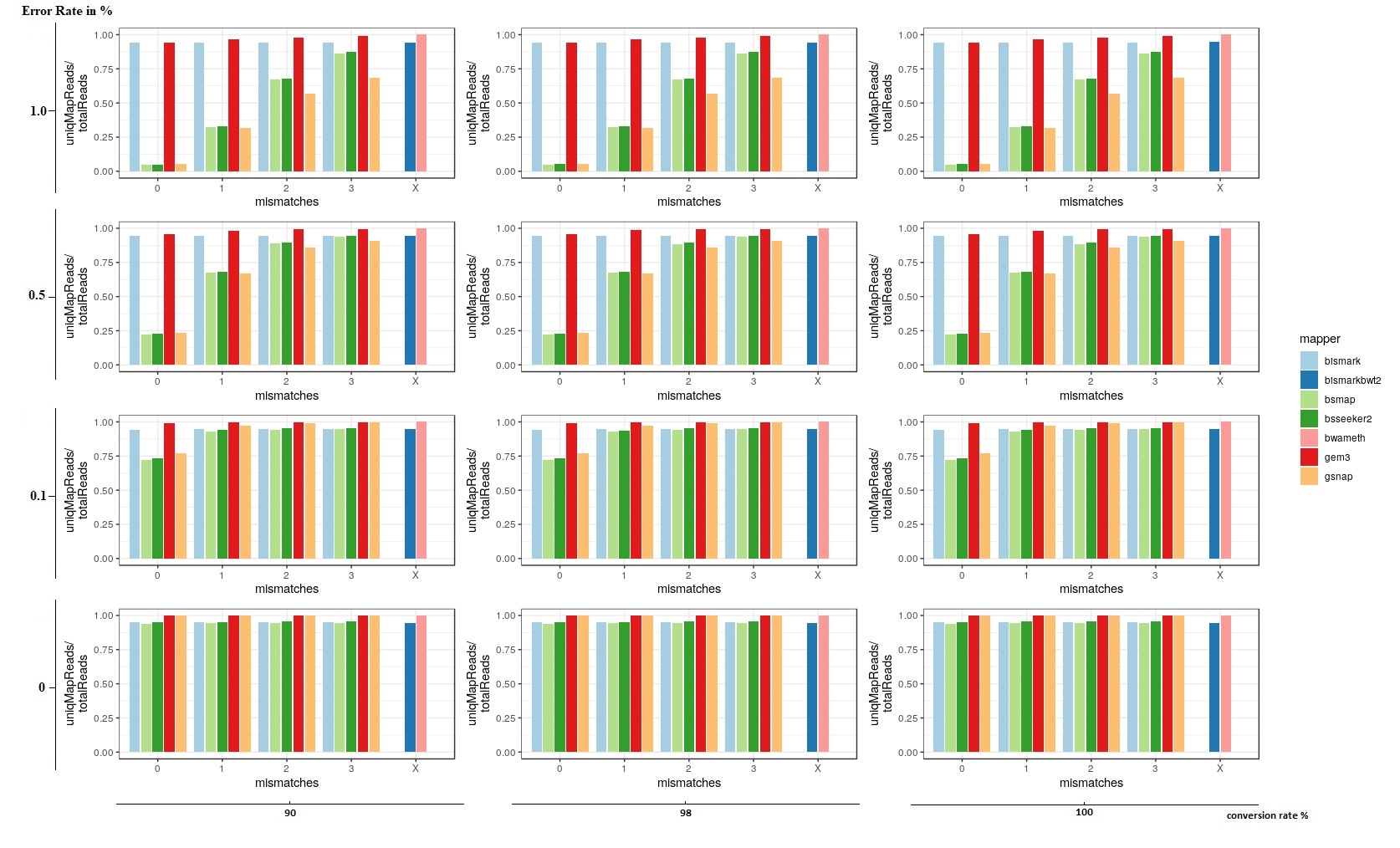


Figure 6: Relative amount of uniquely mapped reads of 7 mappers based on simulated bisulfite sequencing datasets in Glycine max. We simulated the datasets with 4 different error rates [0, 0.1, 0.5 and 1 %] and three different conversion rates [90, 98 and 100%] in a 5fold coverage. For 5 out of 7 mappers we had the opportunity to allow for different numbers of mismatches [0, 1, 2, 3]. Two mappers, bismark using bowtie2 and bwameth, did not allow the adjustment for different numbers of mismatches in the entire read. They are depicted by [X] on the y-axis.


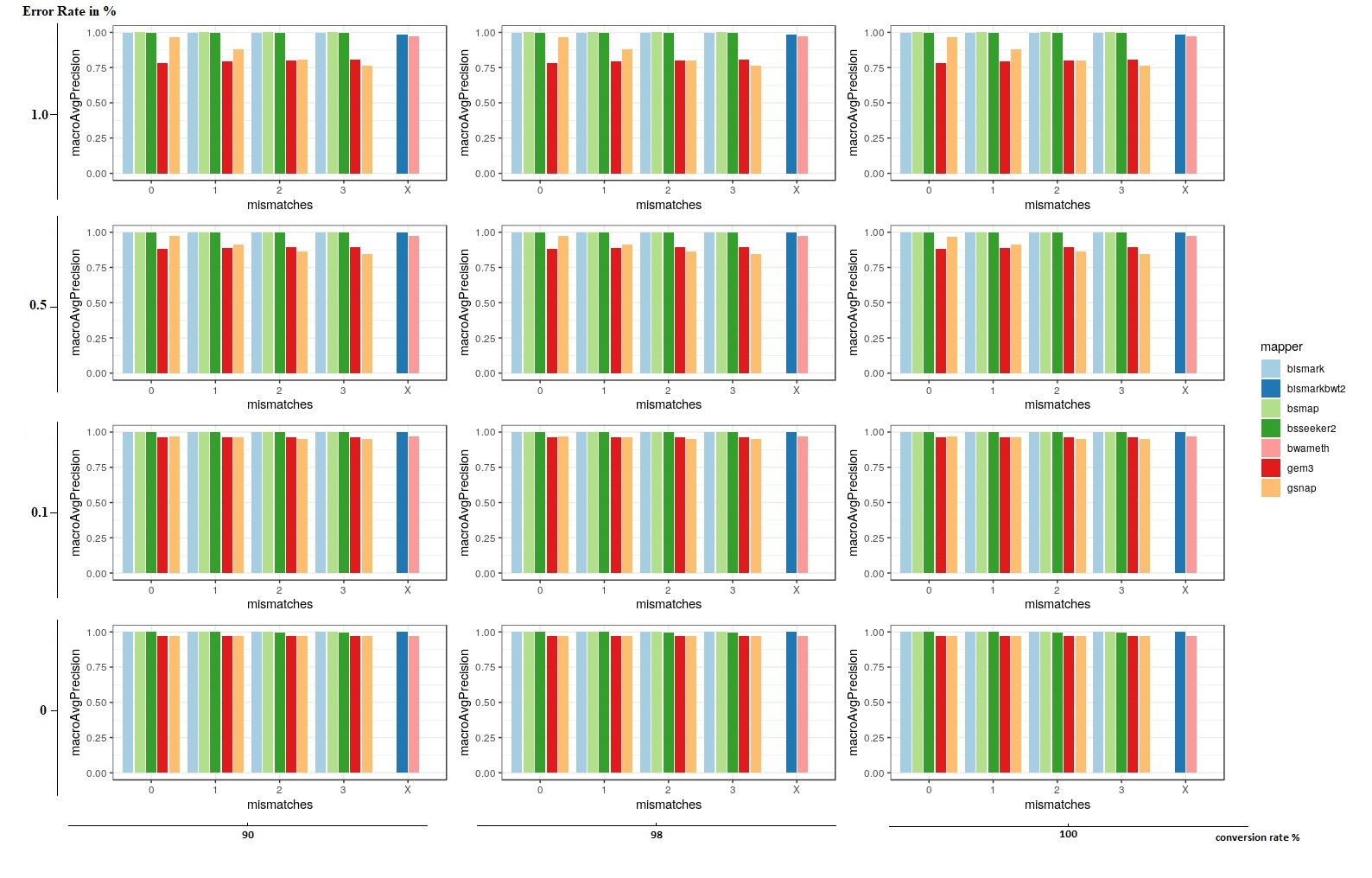


Figure 7: macroAvgPrecision of 7 mappers based on simulated bisulfite sequencing datasets in Solanum tuberosum. We simulated the datasets with 4 different error rates [0, 0.1, 0.5 and 1 %] and three different conversion rates [90, 98 and 100%] in a 5fold coverage. For 5 out of 7 mappers we had the opportunity to allow for different numbers of mismatches [0, 1, 2, 3]. Two mappers, bismark using bowtie2 and bwameth, did not allow the adjustment for different numbers of mismatches in the entire read. They are depicted by [X] on the y-axis.


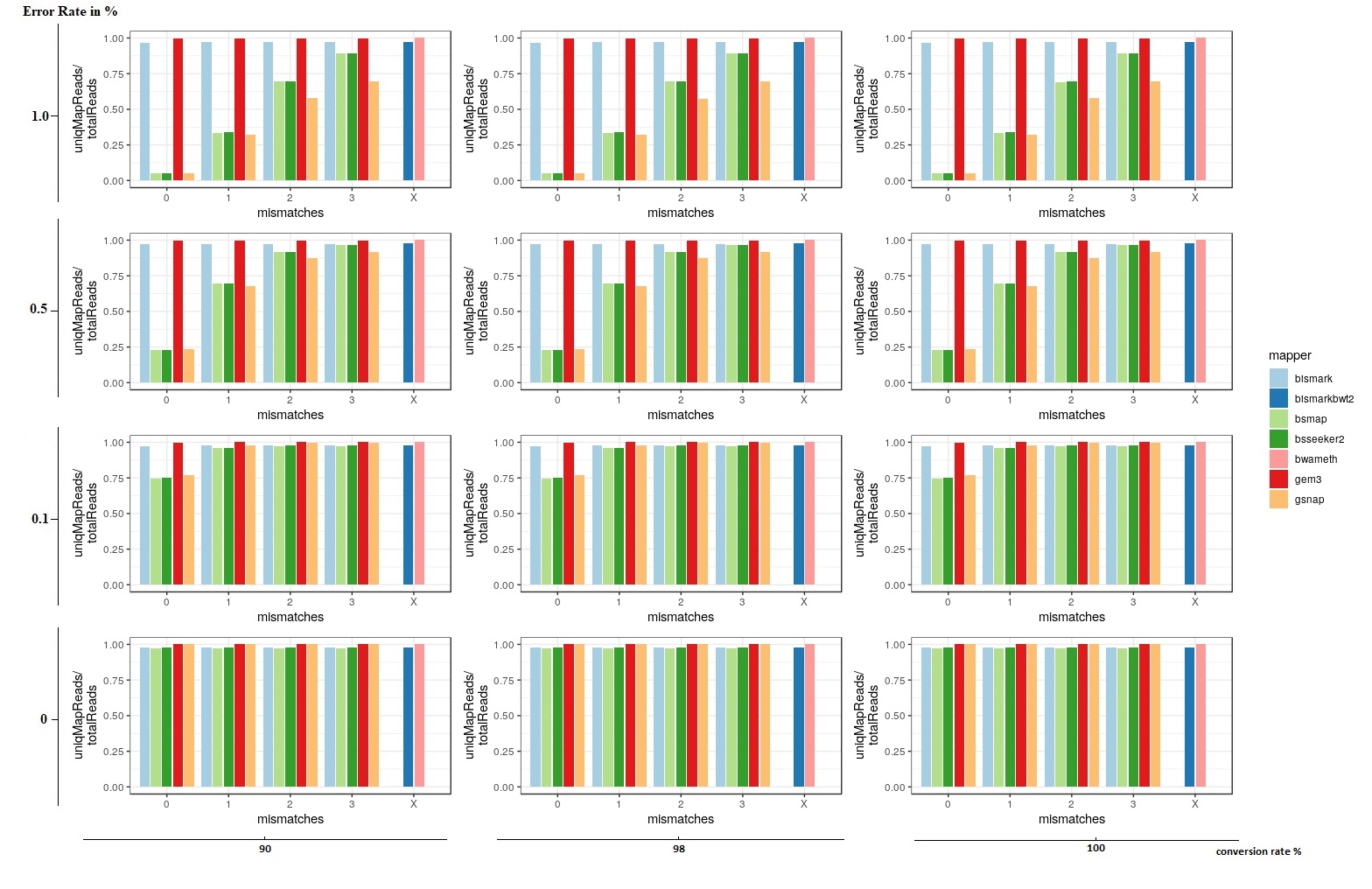


Figure 8: Relative amount of uniquely mapped reads of 7 mappers based on simulated bisulfite sequencing datasets in Solanum tuberosum. We simulated the datasets with 4 different error rates [0, 0.1, 0.5 and 1 %] and three different conversion rates [90, 98 and 100%] in a 5fold coverage. For 5 out of 7 mappers we had the opportunity to allow for different numbers of mismatches [0, 1, 2, 3]. Two mappers, bismark using bowtie2 and bwameth, did not allow the adjustment for different numbers of mismatches in the entire read. They are depicted by [X] on the y-axis.


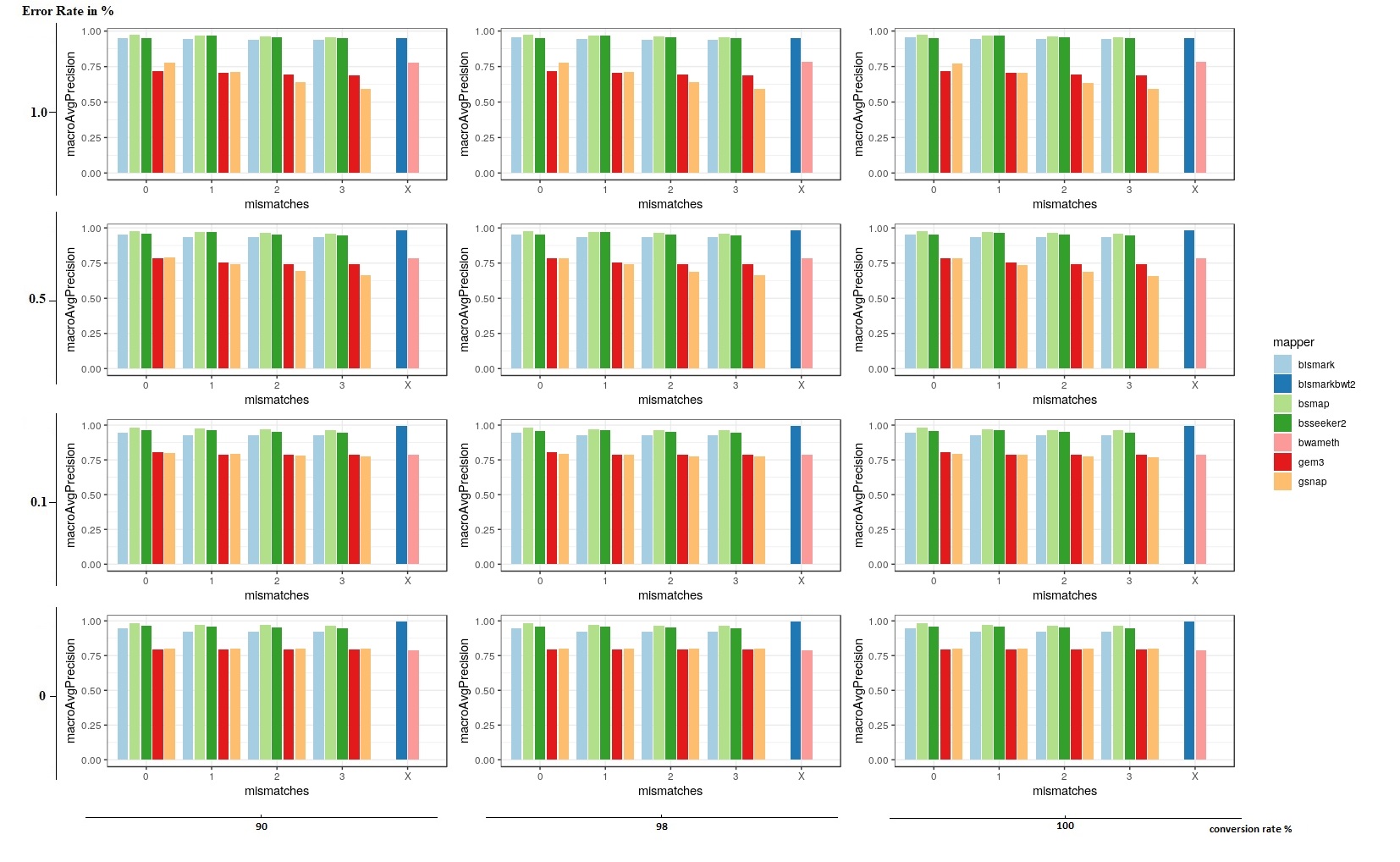


Figure 9: macroAvgPrecision of 7 mappers based on simulated bisulfite sequencing datasets in Zea mays. We simulated the datasets with 4 different error rates [0, 0.1, 0.5 and 1 %] and three different conversion rates [90, 98 and 100%] in a 5fold coverage. For 5 out of 7 mappers we had the opportunity to allow for different numbers of mismatches [0, 1, 2, 3]. Two mappers, bismark using bowtie2 and bwameth, did not allow the adjustment for different numbers of mismatches in the entire read. They are depicted by [X] on the y-axis.


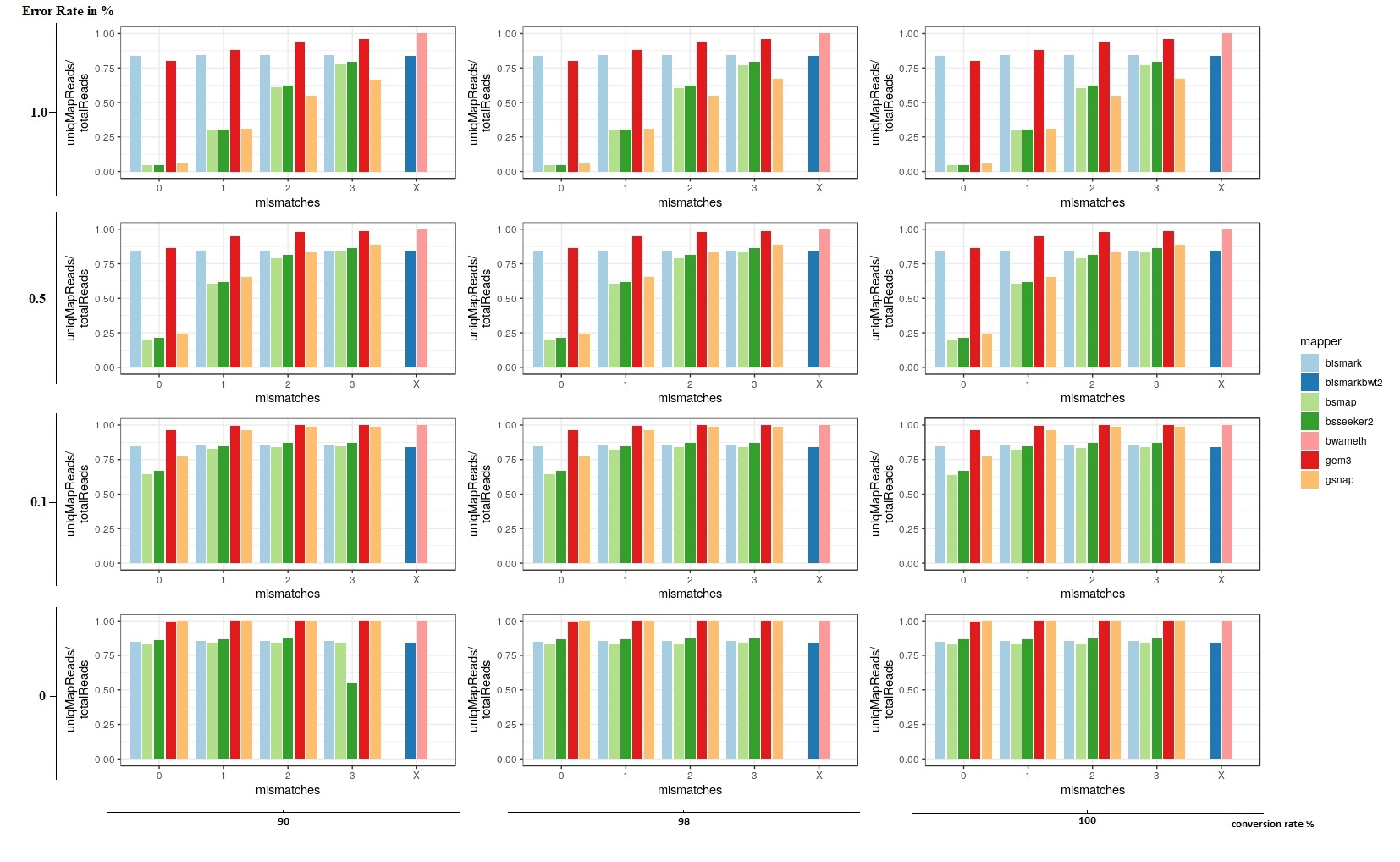


Figure 10: Relative amount of uniquely mapped reads of 7 mappers based on simulated bisulfite sequencing datasets in Zea mays. We simulated the datasets with 4 different error rates [0, 0.1, 0.5 and 1 %] and three different conversion rates [90, 98 and 100%] in a 5fold coverage. For 5 out of 7 mappers we had the opportunity to allow for different numbers of mismatches [0, 1, 2, 3]. Two mappers, bismark using bowtie2 and bwameth, did not allow the adjustment for different numbers of mismatches in the entire read. They are depicted by [X] on the y-axis.
